# Fluorescent probes for monitoring myeloperoxidase-derived hypochlorous acid: a comparative study

**DOI:** 10.1038/s41598-022-13317-8

**Published:** 2022-06-03

**Authors:** Karolina Pierzchała, Marlena Pięta, Monika Rola, Małgorzata Świerczyńska, Angelika Artelska, Karolina Dębowska, Radosław Podsiadły, Jakub Pięta, Jacek Zielonka, Adam Sikora, Andrzej Marcinek, Radosław Michalski

**Affiliations:** 1 Department of Chemistry, Institute of Applied Radiation Chemistry, Lodz University of Technology, Zeromskiego 116, 90-924 Lodz, Poland; 2 Department of Chemistry, Institute of Polymer and Dye Technology, Lodz University of Technology, Stefanowskiego 12/16, 90-924 Lodz, Poland; 3 Department of Biophysics and Free Radical Research Center, Medical College of Wisconsin, 8701 Watertown Plank Road, Milwaukee, WI 53226 USA

**Keywords:** Fluorescent probes, Photobiology, Bioanalytical chemistry

## Abstract

MPO-derived oxidants including HOCl contribute to tissue damage and the initiation and propagation of inflammatory diseases. The search for small molecule inhibitors of myeloperoxidase, as molecular tools and potential drugs, requires the application of high throughput screening assays based on monitoring the activity of myeloperoxidase. In this study, we have compared three classes of fluorescent probes for monitoring myeloperoxidase-derived hypochlorous acid, including boronate-, aminophenyl- and thiol-based fluorogenic probes and we show that all three classes of probes are suitable for this purpose. However, probes based on the coumarin fluorophore turned out to be not reliable indicators of the inhibitors’ potency. We have also determined the rate constants of the reaction between HOCl and the probes and they are equal to 1.8 × 10^4^ M^−1^s^−1^ for coumarin boronic acid (CBA), 1.1 × 10^4^ M^−1^s^−1^ for fluorescein based boronic acid (FLBA), 3.1 × 10^4^ M^−1^s^−1^ for 7-(*p*-aminophenyl)-coumarin (APC), 1.6 × 10^4^ M^−1^s^−1^ for 3’-(*p*-aminophenyl)-fluorescein (APF), and 1 × 10^7^ M^−1^s^−1^ for 4-thiomorpholino-7-nitrobenz-2-oxa-1,3-diazole (NBD-TM). The high reaction rate constant of NBD-TM with HOCl makes this probe the most reliable tool to monitor HOCl formation in the presence of compounds showing HOCl-scavenging activity.

## Introduction

**Figure 1:**
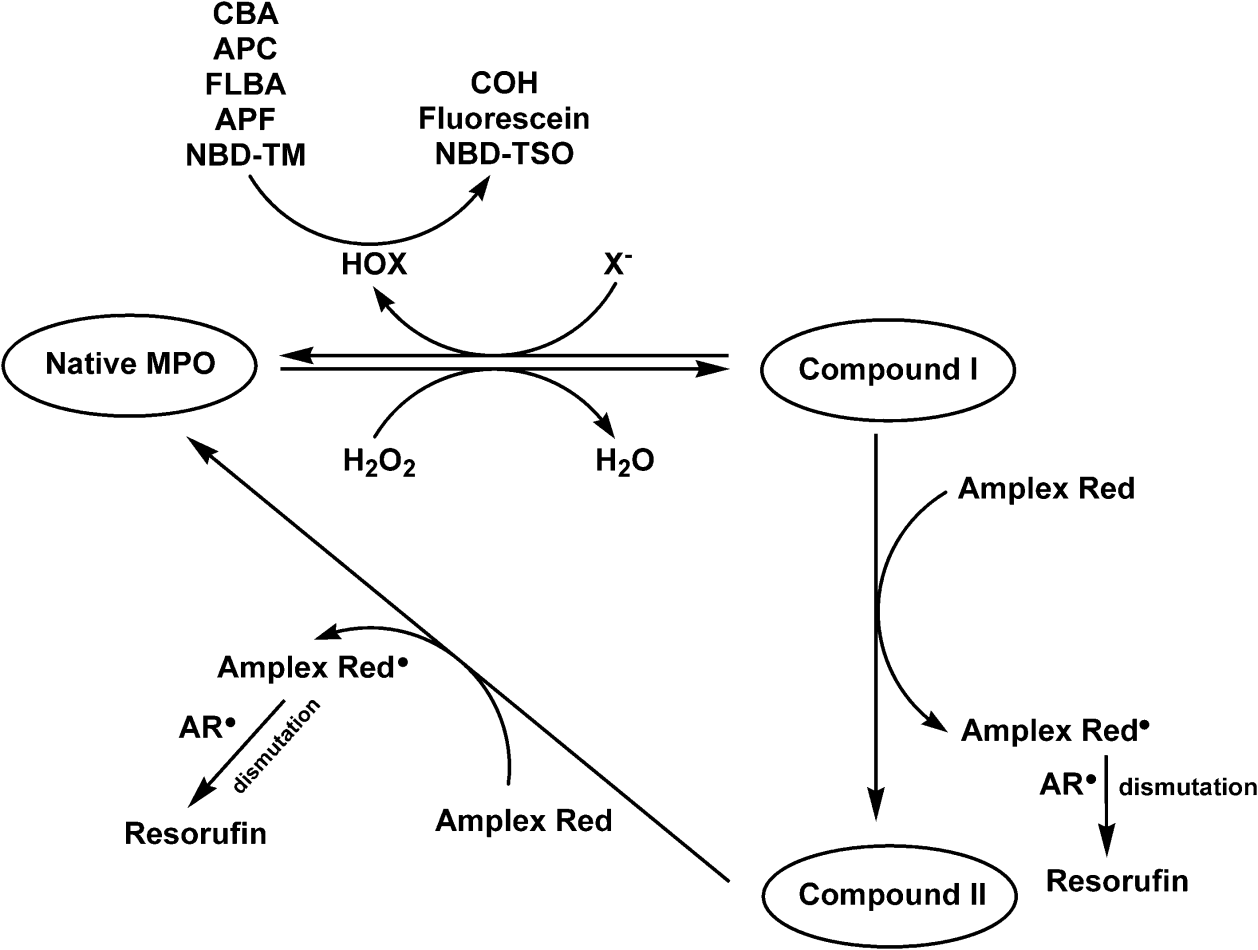
Redox cycles of MPO and methods of their monitoring.

Myeloperoxidase (MPO) is a mammalian heme peroxidase that produces hypohalous acids (HOX, where X is a halogen atom, Fig. 1), e.g. hypochlorous acid (HOCl), in the presence of halides (X^–^) and hydrogen peroxide (H_2_O_2_). Hypohalous acids are powerful oxidants and are crucial reactive species that immune cells use to eradicate invading pathogens. Nonetheless, excessive or misplaced formation of HOCl and/or other hypohalous acids is associated with inflammatory pathologies^1,2^. Strong association of halogenating activity of MPO with initiation and propagation of inflammatory diseases resulted in the interest in developing small molecule inhibitors of the MPO enzyme as potential drugs and protective agents in various pathologies involving an inflammatory component^3^. While several compounds interfering with the peroxidase activity of MPO (e.g. nitroxides, tyrosine-containing peptides, hydroxamic acids, indoles, tryptamines, acetaminophen and other anti-inflammatory drugs)^4–10^ showed protective effects in various animal models of inflammation^11–14^, there is a need for more potent inhibitors, which would irreversibly block HOCl generation by the enzyme. The search of potent MPO inhibitors demands application of high throughput screening (HTS)-compatible rigorous methodology(ies) to detect and quantify MPO-derived HOCl, with the aim to screen large libraries of compounds.

Numerous probes and detection approaches have been developed over the last decade to measure the production of HOCl by MPO^15–21^. Historically, the most often used methods involves chlorination of primary amines, e.g. taurine^6,15^. In this method, the produced taurine chloramine is quantified by measuring the oxidation of 5-thio-2-nitrobenzoic acid (TNB) to a colorless product^22^ or by oxidation of 3,3',5,5'-tetramethylbenzidine (TMB) in the presence of potassium iodide, to a strongly absorbing blue product^23^. Another compound used for this purpose is tyrosine which forms in the reaction with HOCl 3-chlorotyrosine that can be subsequently quantified by HPLC or LC/MS^15,24,25^. Those methodologies are, however, relatively complex and/or not compatible with HTS. Most HTS assays are based on reactions yielding easily detectable luminescent (fluorescent, chemiluminescent or bioluminescent) products, due to high detection sensitivity, and compatibility with multiwell plate readers.

Recent progress in the development and use of profluorescent probes for detection of reactive oxygen species enabled specific detection of various oxidants of interest^16,17,26–35^. While the number of recent reports on the development of fluorescent probes for HOCl is relatively high, comparing with probes for other cellular oxidants, only few classes of probes gained wider interest and application, due to their reliability and commercial availability. Those include probes containing thiols, aminophenyl and boronate groups as the sensing moieties for HOCl. It has been shown that boronate probes react with peroxynitrite (ONOO^‒^), amino acid hydroperoxides, and H_2_O_2_ with the rate constants of 10^6^, 10^1^, 10^0^ M^−1^s^−1^ order of magnitude, respectively^36–38^. We have also shown that simple aromatic boronates react directly and stoichiometrically (1:1 ratio) with HOCl yielding the corresponding phenolic derivatives with the second-order rate constant of 10^4^ M^−1^s^−1^ order of magnitude^38^. *O*-substituted *p*-aminophenyl derivative of fluorescein has been shown to release fluorescein upon reaction with HOCl, while it also respond to HRP/H_2_O_2_, ^•^OH and ONOO^–^-derived radicals^15,39,40^. The kinetic data for this class of probes are, however, very limited. The third class of probes is based on a rapid reaction of a thiol group with HOCl, producing a sulfenyl chloride intermediate, which undergoes further reactions to produce a fluorescent product, via a mechanism depending on the chemical design of the probe^20,41–43^.

**Figure 2:**
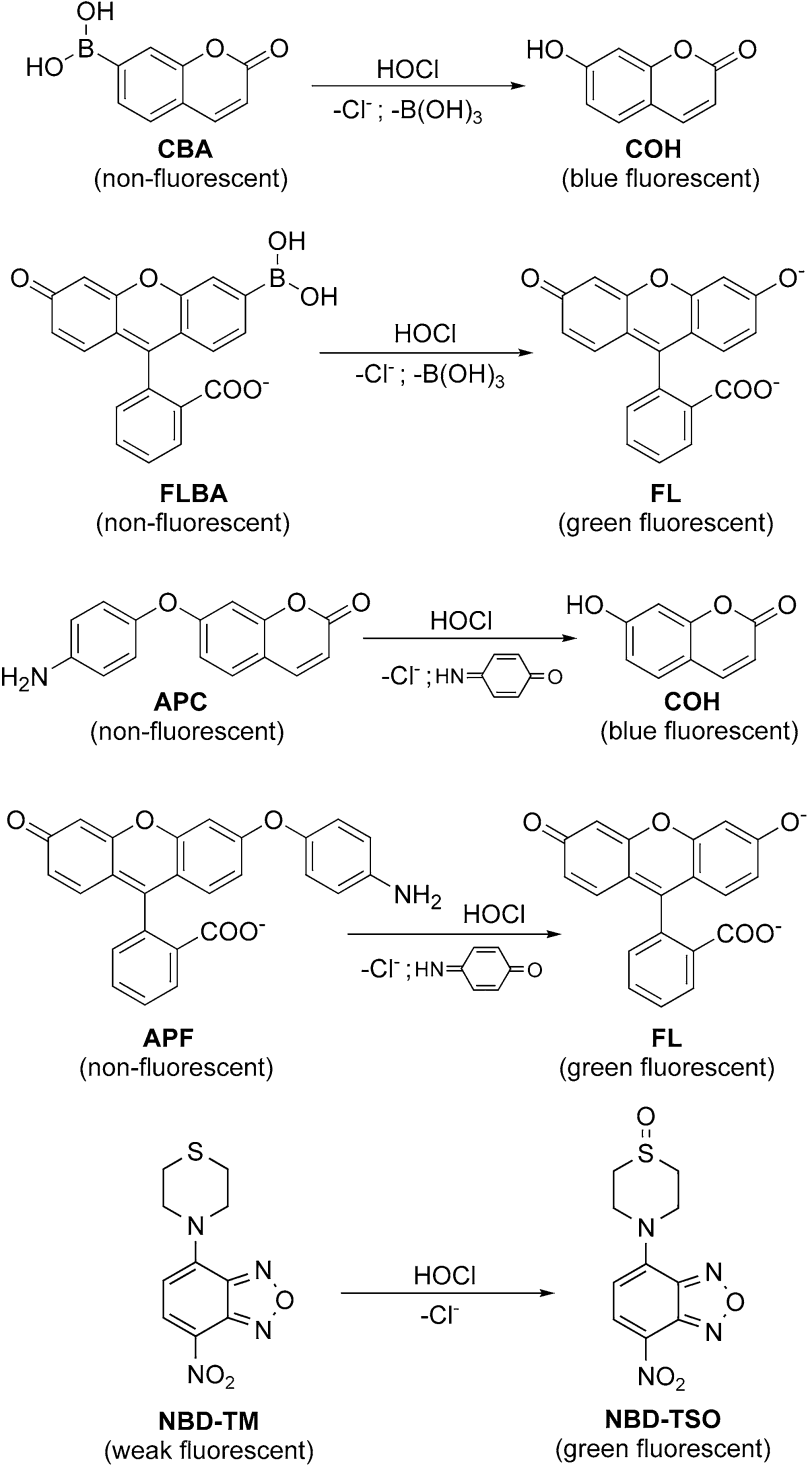
Structures of the investigated probes and their oxidation products.

In this work, we characterized and compared the HOCl detection performance of the boronate-, aminophenyl- and thiol-based fluorogenic probes, with special emphasis on their applicability for HTS-compatible screening for MPO inhibitors. The probes used include coumarin boronic acid (CBA), fluorescein boronic acid (FLBA), 3’-(*p*-aminophenyl)-fluorescein (APF), 7-(*p*-aminophenyl)-coumarin (APC), and the newly developed thiol based probe, 4-thiomorpholino-7-nitrobenz-2-oxa-1,3-diazole (NBD-TM) (Fig. 2)^44^. For all tested probes, the rate constants with HOCl and the stability of produced fluorophores in the enzymatic system containing MPO/H_2_O_2_/Cl^─^ were determined. The ability of the probes to identify and characterize MPO inhibitors was validated with the use of established MPO inhibitors, i.e. 4-aminobenzoic acid hydrazide (ABAH) and sodium azide. The obtained IC_50_ values by means of CBA, FLBA, APC, APF, and NBD-TM probes were compared to the IC_50_ values for these inhibitors determined using the Amplex Red based peroxidative assay. Finally, the Z’ parameter reflecting the quality of the assay was determined for each probe.

## Results

### Monitoring MPO-derived HOCl

**Figure 3:**
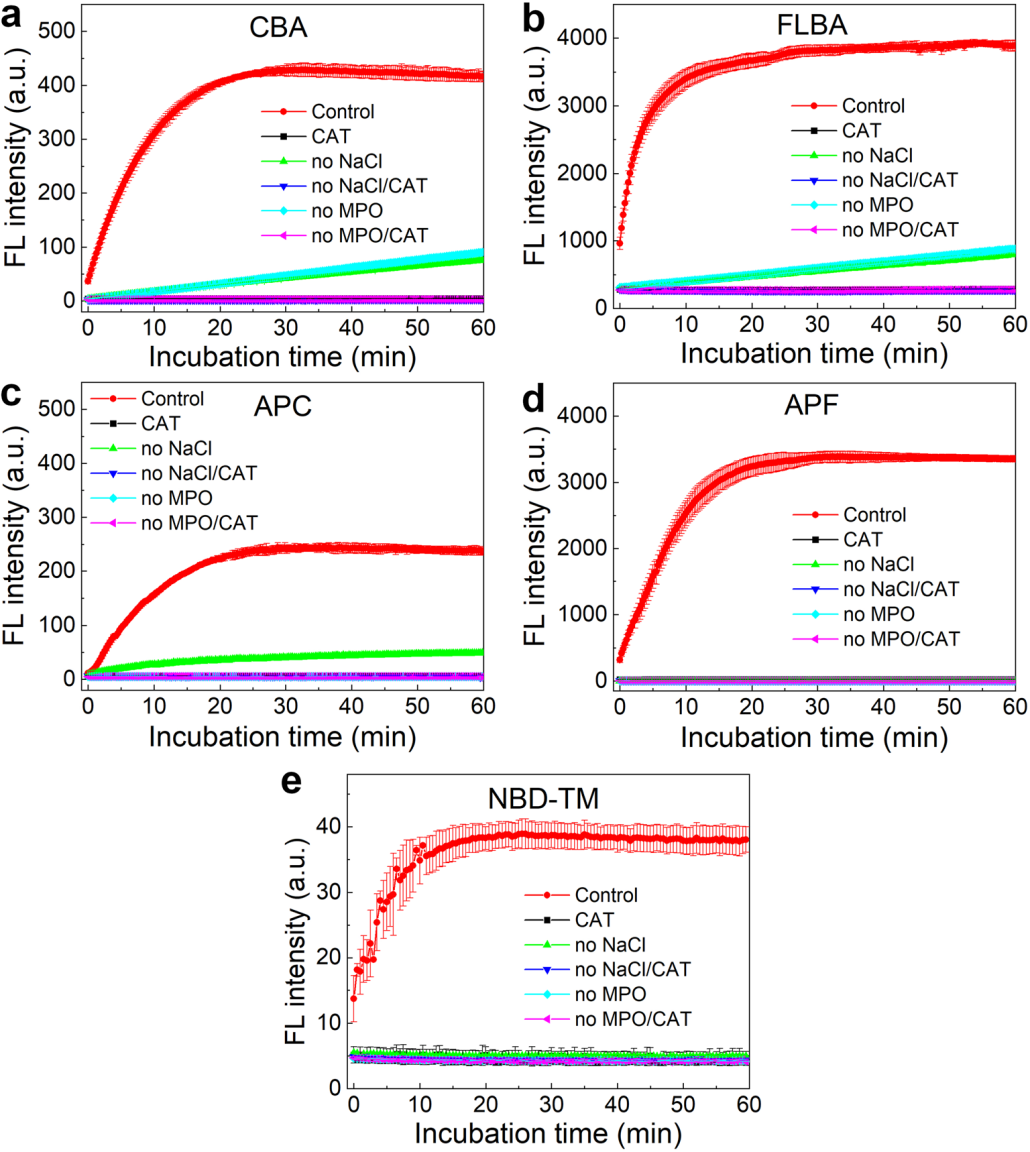
(**a**) Oxidation of CBA to COH in the incubation mixtures containing CBA (20 µM), MPO (1.2 nM), H_2_O_2_ (10 µM), NaCl (0.1 M), and phosphate buffer (50 mM, pH 7.4) (red); in the presence of catalase (100 U/ml, black); in the absence of NaCl (green); in the absence of NaCl but in the presence of catalase (dark blue); in the absence of MPO (light blue); in the absence of MPO but in the presence of catalase (pink). (**b**–**e**) same as (**a**) but instead of CBA, incubation mixtures contained FLBA, APC, APF, and NBD-TM, respectively, and were oxidized to appropriate fluorescent compounds. (Each panel is a representative result of three independent experiments. Points represent means ± S.D.)

In the first set of experiments, the performance of the boronate probes for monitoring of MPO-mediated HOCl production was determined. The CBA probe (20 µM) was incubated in the mixture containing MPO (1.2 nM), low concentration of H_2_O_2_ (10 µM), NaCl (100 mM) and phosphate buffer (50 mM, pH 7.4) (Fig. 3a). During the incubation the increase in fluorescence intensity at 450 nm was observed, with almost complete oxidation observed within 20 min of incubation. This signal was ascribed to the formation of 7-hydroxycoumarin, the fluorescent product of CBA oxidation (Fig. 2), on the basis of fluorescence spectra^32^ and of known reactivity of boronate-based probes towards HOCl^29,36,38,45^. In order to confirm that the observed fluorescence signal is due to the oxidation of CBA probe by HOCl and not by reaction with H_2_O_2_, CBA was incubated under similar conditions but in the absence of MPO. Under these conditions, CBA was oxidized by H_2_O_2_ very slowly and the production of COH over the first 20 min of incubation was negligible. The similar result was obtained in the absence of NaCl. The presence of catalase (100 U/ml) in the incubation mixtures completely quenched the fluorescence signal regardless of the composition of a sample.

The analogical experiment was performed using fluorescein boronic acid (FLBA) with similar set of incubation mixtures (Fig. 3b). In this case, FLBA was also oxidized by MPO-derived HOCl to the fluorescent product with the oxidation completed within 20 min of incubation (Fig. 2). The formation of fluorescein (FL) was confirmed by comparing the measured absorption and emission spectra with the spectra of an original standard of fluorescein. Similarly, to the CBA probe, FLBA was also slowly oxidized by H_2_O_2_ unless catalase was present in the reaction mixture. Both boronate probes were oxidized in the MPO/H_2_O_2_/Cl^–^ system (Fig. 3a,b). While the increase in a fluorescent signal reached plateau for the incubation time about 20–30 min, the addition of another amount of H_2_O_2_ resulted in further oxidation of the probes indicating that H_2_O_2_ was the limiting substrate in the reaction mixtures (not shown).

Another class of probes which were tested in the MPO/H_2_O_2_/Cl^–^ system were *p*-aminophenyl derivatives of fluorescein and 7-hydroxycoumarin: 3’-(*p*-aminophenyl)fluorescein (APF) and 7-(*p*-aminophenyl)coumarin (APC), respectively (Fig. 2). These probes undergo *O*-dearylation in the reaction with HOCl (Fig. 2). APF was oxidized to fluorescein only when both substrates, Cl^–^ and H_2_O_2_, and the enzyme were present in the sample (Fig. 3d). The absence of any component or the addition of catalase completely blocked the formation of fluorescein (Fig. 3d). The APC probe was also oxidized by MPO-derived HOCl (Fig. 3c). Nonetheless, APC also proved to be a substrate in the peroxidation cycle of MPO. Incubation of APC in the sample containing MPO and H_2_O_2_, but not NaCl, led to a slow increase in fluorescence intensity, indicating the formation of the COH product. APC was not oxidized directly by H_2_O_2_ and the presence of catalase inhibited the signal (Fig. 3c).

The third class of detectors tested in the MPO/H_2_O_2_/Cl^–^ system is based on HOCl-mediated thiol oxidation, and is represented in this study by the NBD-TM probe (Fig. 2). This probe in the presence of MPO, H_2_O_2_, and Cl^–^ is oxidized to the appropriate fluorescent thiomorpholine-S-oxide (NBD-TSO) (Fig. 3e). Similar to the APF probe, the lack of any substrate for MPO or the presence of catalase fully inhibited any increase in the fluorescence signal (Fig. 3e).

To check the stability of the COH, FL, and NBD-TSO fluorescence signal under the oxidizing conditions of MPO/H_2_O_2_/Cl^–^, the fluorophores were incubated under the same reaction conditions, as described above for the five probes used in the study (Supplementary Figure S1). In the presence of MPO, H_2_O_2_, and NaCl, the fluorescence intensity of COH was decreasing with the time of incubation (Supplementary Figure S1). Similar result was observed for the incubation containing MPO and H_2_O_2_ but the kinetic traces of COH decomposition for both samples were different, indicating two different processes. For the sample containing MPO/H_2_O_2_/Cl^–^ the decrease of COH concentration was assigned to the chlorination of COH, while for the sample that did not contain NaCl, COH was oxidized by MPO via its peroxidatic activity. The changes in the fluorescence intensity of fluorescein were less pronounced than COH under the same incubation conditions, and only in the sample containing MPO/H_2_O_2_/Cl^–^, a slight decrease in the fluorescence intensity was observed (Supplementary Figure S1), which we assign to fluorescein reaction with HOCl. In turn, NBD-TSO was slowly decomposing or precipitated during the time of incubation, regardless of its composition (Supplementary Figure S1). Overall, the fluorophores formed from the probes used may undergo consumption in the presence of MPO, H_2_O_2_ and Cl^–^, due to their reaction with HOCl and/or via peroxidatic activity of MPO in the absence of Cl^–^. This is, however, expected to occur only under the condition of excess of the oxidants (H_2_O_2_ and/or HOCl), as evidenced by the stable fluorescence signals of the products observed within 1 h of incubation (Fig. 3). The experimental design for monitoring HOCl involving probes producing such fluorophores should, therefore, take into account potential consumption of the fluorescent product, when the amount of the oxidant exceeds the probe availability.

### Rate constants with HOCl

**Figure 4:**
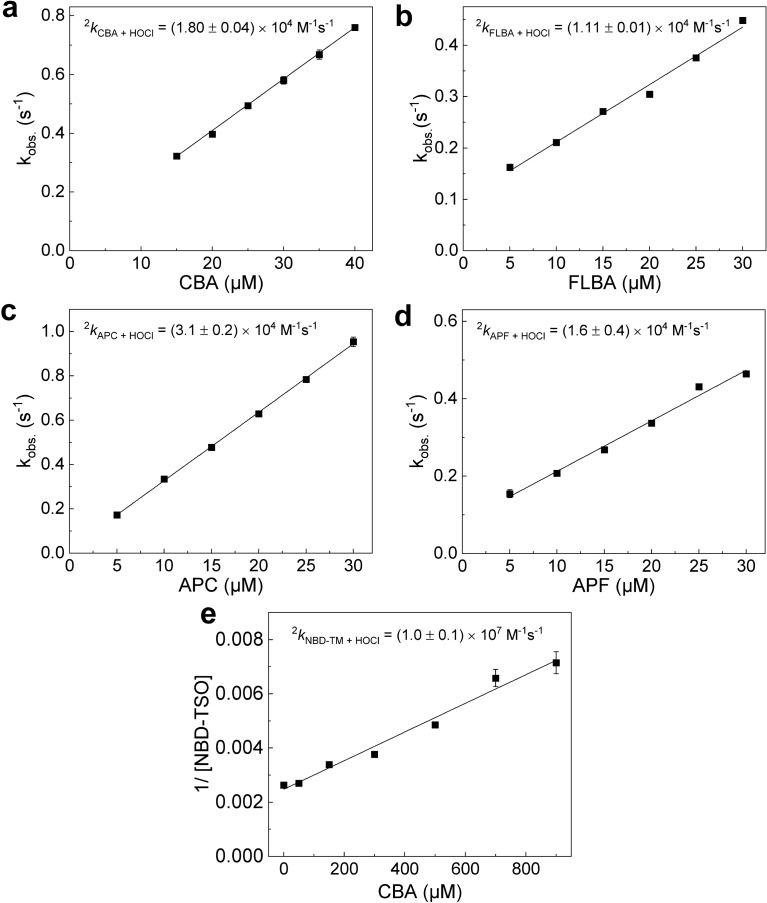
(**a**) The dependences of the pseudo-first-order rate constants of the reaction between CBA and HOCl on the initial concentration of CBA. Reaction mixtures contained 1 µM HOCl, 50 mM phosphate buffer (pH 7.4), 15–40 µM CBA. (**b**), (**c**), and (**d**) same as (**a**) but incubation mixtures contained FLBA (5–30 µM), or APC (5–30 µM), or APF (5–30 µM), instead of CBA. (**e**) The relationship used to determine the rate constant between HOCl and NBD-TM. The solid line in the panel (**e**) represents the linear fitting according to the Eq. (4). Incubation mixtures contained 50 mM phosphate buffer (pH 7.4), 0.5 µM HOCl, 0–900 µM CBA, and 1.5 µM NBD-TM. The R-square parameter for the linear fittings was at least 0.98 or higher. The observed non-zero intercepts are attributed to the slow decay of HOCl in the presence of organic solvent. (Each panel is a representative result of three independent experiments, points represent means ± S.D.; rate constants were calculated on the basis of three independent experiments and are given as mean ± S.D.).

One of the crucial parameters controlling the performance of any probe used for the detection of chlorinating activity of MPO is the rate constant of the reaction between the probe and HOCl. For the boronate probes, and *O*-substituted *p*-aminophenyl derivatives of fluorescein and coumarin, the rate constants with HOCl were measured directly by monitoring the kinetics of formation of the fluorescent products, using a stopped-flow spectrometer. The concentrations of the probes used in the experiments were at least five times higher than that of HOCl (Fig. 4a–d). Due to high rate constant and rapid reaction completion, in the case of NBD-TM probe, the rate constant was measured using the competition kinetic methodology combined with LC/MS-based detection of the reaction products (Fig. 4e). In this experiment CBA was used as a reference compound.

The rate constants of 1.8 × 10^4^ M^−1^s^−1^ and 1.1 × 10^4^ M^−1^s^−1^ were obtained for CBA and FLBA, respectively, which are typical values reported for this class of probes (Fig. 4a,b)^29,36,38,45^. The measured rate constants for APC and APF were slightly higher than those determined for CBA and FLBA and were equal to 3.1 × 10^4^ M^−1^s^−1^ and 1.6 × 10^4^ M^−1^s^−1^, respectively (Fig. 4b,c). Additionally, for APC and APF nearly identical rate constants were determined by competition kinetics (Supplementary Figure S2). The highest rate constant was obtained for the NBD-TM probe which was equal to 1.0 × 10^7^ M^−1^s^−1^. This rate constant was calculated using a second order rate constant of 1.8 × 10^4^ M^−1^s^−1^ for the reference compound (CBA). The determined value of the rate constant for NBD-TM is consistent with the reaction rate constants reported for other thioethers with HOCl^41,46^.

### Application of the probes to determine the potency of MPO inhibitors

**Figure 5:**
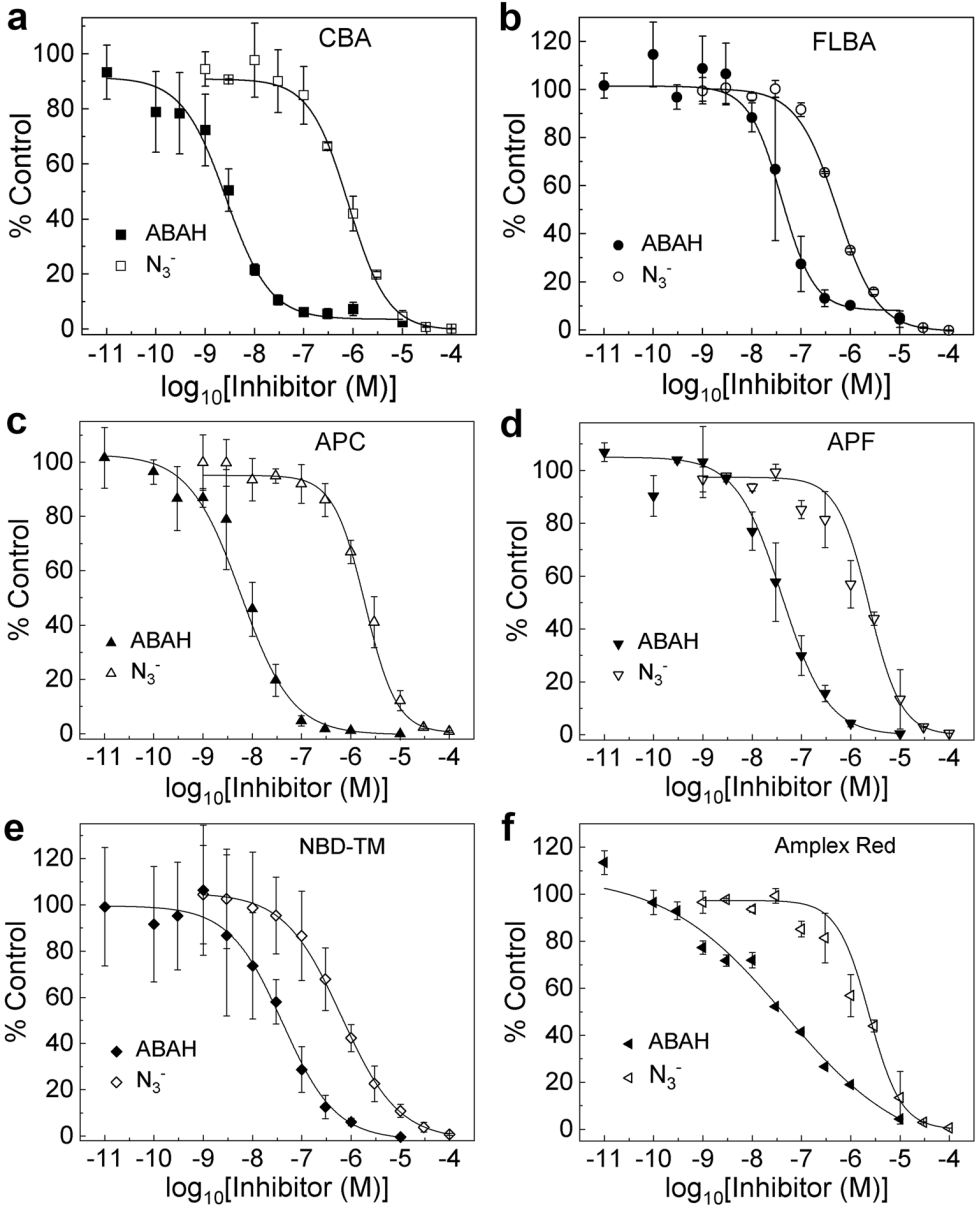
(**a**) Dose–response curve for ABAH and azide anion determined by CBA assay. (**b**–**e**) same as (**a**) but instead of CBA the FLBA, APC, APF, and NBD-TM probes have been used, respectively. Mixtures contained MPO (1.2 nM), H_2_O_2_ (10 µM), NaCl (0.1 M), phosphate buffer (50 mM, pH 7.4), and the appropriate probe (20 µM). (**f**) Dose–response curves for azide anion and ABAH determined by peroxidative activity assay. Mixture contained Amplex Red (20 µM), MPO (1.2 nM), H_2_O_2_ (10 µM), phosphate buffer (50 mM, pH 7.4), and azide anion or ABAH, but no NaCl. In the calculations the initial rates of probes oxidation were used. (Data are means ± standard deviation of three independent experiments).

To establish the applicability of the probes to screen for novel MPO inhibitors, all the probes under study were used to determine the half-maximal inhibitory concentration (IC_50_) values for two well-established MPO inhibitors: 4-aminobenzoic hydrazide (ABAH) and the azide anion (N_3_^–^). The dose–response curves for ABAH and N_3_^–^ are presented in Fig. 5 and the determined IC_50_ values are shown in Table 1. The IC_50_ values for ABAH are in the range of 2.6 to 40.6 nM and for the azide ion are between 0.5 and 2.4 µM. In the case of the ABAH inhibitor, the CBA and APC probes yielded significantly lower IC_50_ values than the other three probes. As both probes share the same oxidation product, COH, these discrepancies can be attributed to the interference of COH with the mechanism of MPO inhibition by ABAH. Therefore, we recommend the IC_50_ value of 40 nM, obtained consistently for all four other probes used. In the case of the azide anion the discrepancies between the IC_50_ values for different probes were less noticeable. While similar values were obtained using boronate and the NBD-TM probes, more than two-fold higher IC_50_ values were determined for the probes that undergo *o*-dearylation, APC and APF (Table 1). Our tentative explanation for those explanation is potential oxidation of those two probes, together with Amplex Red (see below) by the azidyl radical (N_3_^•^), to produce the fluorescent products. Azidyl radical is known to be formed in the presence of MPO, H_2_O_2_ and azide^47^. Therefore, we recommend the average IC_50_ value of 0.7 µM, obtained from the three probes resistant to one-electron oxidation by the N_3_^•^ radical: CBA, FLBA and NBD-TM. In order to ensure the accurate data interpretation, the competition experiment between the inhibitors and the probes for HOCl was performed and the effect of the inhibitors on the fluorescence intensity of the products formed (fluorophores) was examined. At a 20 µM concentration of the probes their reaction with HOCl and the observed fluorescence intensities were not influenced by the inhibitors in the concentration range used.

**Table 1:** IC50 values for ABAH and azide anion determined by means of the CBA, FLBA, APC, APF, and NBD-TM probes.

Assay/probe	ABAH IC_50_ (nM)	Azide anion IC_50_ (µM)
CBA	2.64	0.81
FLBA	39.98	0.54
APC	5.86	1.94
APF	40.33	2.37
NBD-TM	40.56	0.63
Amplex red	47.45	1.04

Next, the potency of both MPO inhibitors was tested in a peroxidative activity assay. For this purpose, the Amplex Red probe was used. Amplex Red undergoes oxidation to fluorescent resorufin in the presence of H_2_O_2_ and peroxidases (Fig. 1), and the exact mechanism of its one-electron (peroxidatic) oxidation was recently reported^48^. For monitoring the peroxidatic activity of MPO, Amplex Red was incubated with the enzyme and H_2_O_2_, but in the absence of NaCl. The constructed dose–response curves are shown in Fig. 5f). The measured IC_50_ values for ABAH and N_3_^–^ were equal to 48 nM and 1.0 µM (Table 1), respectively, and were close to their IC_50_ values determined using the probes for monitoring MPO chlorination activity.

### Z’ values

The Z’ parameter is a measure of the assay quality in terms of its applicability to screen for bioactive compounds in cell-free or cell-based systems. The Z’ value of 0.5 or higher is generally accepted for the application of any assay for high throughput screening (HTS) of large chemical libraries. For the probes tested, the Z’ parameter was calculated on the basis of end-point measurements (fluorescence measured at a specific time of incubation) and kinetic measurements (rates of fluorescence increase), according to the Eq. 5. In the end-point measurements the Z’ values were calculated for the time of incubation equal to 20 min. To calculate the kinetic Z’ the initial rates of fluorescent product formation over 5 min were used. The determined Z’ values were around 0.9 for CBA, FLBA, APC, and APF, both for the end-point and kinetic measurements. The lowest Z’ values around 0.7 were obtained for the NBD-TM probe. All the probes produced the Z’ values compatible with HTS applications.

### Effect of DMSO on the probes’ performance

**Figure 6:**
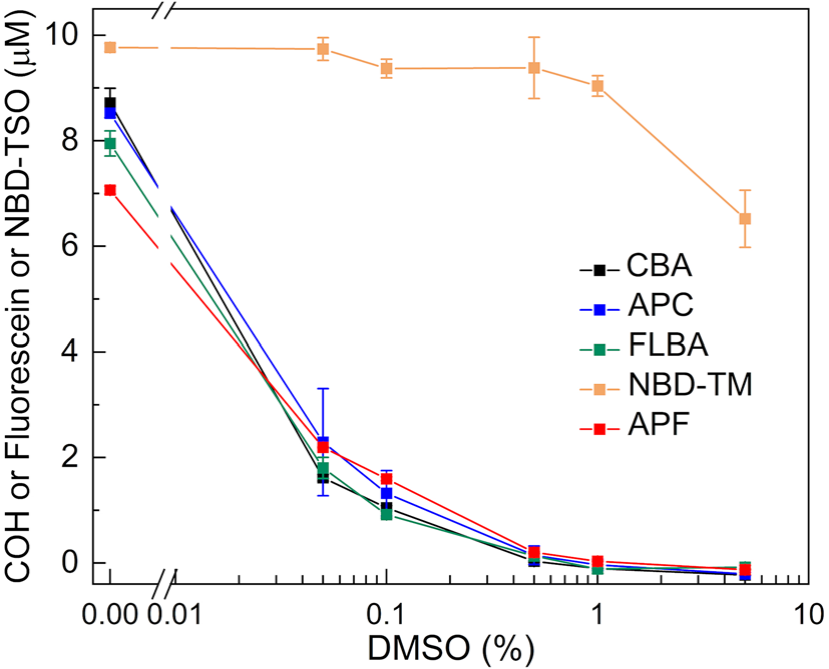
Effect of DMSO on detected HOCl concentrations by the CBA, APC, APF, FLBA, and NBD-TM probes. Incubation mixtures contained 20 µM of a probe, 10 µM HOCl, 0–5% v/v (0–0.7 M) DMSO, and 50 mM phosphate buffer (pH 7.4). (Representative result of three independent experiments is shown. Points represent means ± S.D.)

DMSO is a solvent well tolerated by cells and it is often used to dissolve organic compounds intended for cell culture studies. Most chemical libraries for HTS applications use DMSO as the solvent. However, DMSO reacts directly with HOCl with a second-order rate constant of 350 ± 40 M^−1^s^−1^ and it has been shown that it may interfere with oxidation of boronate probes by HOCl^49^. Thus, the influence of DMSO additive on the observed fluorescence signal generated in the reaction between HOCl and the studied probes was evaluated. In this experiment, the incubation mixtures contained the chosen probe (20 µM), phosphate buffer (50 mM, pH 7.4), and DMSO in the amounts between 0–5% v/v (0–0.7 M). The reaction with HOCl was initiated by the bolus addition of 1 mM sodium hypochlorite to obtain the final HOCl concentration of 10 µM. In the case of NBD-TM that reacts rapidly and stoichiometrically (1:1 ratio) with HOCl^44^ about 10 µM of NBD-TSO was formed (Fig. 6). In case the CBA, FLBA, APC, and APF probes, a slightly lower yields of the products were observed, possibly due to their lower reactivity towards HOCl and to scavenging of HOCl by acetonitrile^29^ that was used to prepare the probes stock solutions. The presence of 0.05% (7 mM) or 0.1% v/v (14 mM) DMSO had negligible effect on the HOCl-induced oxidation of NBD-TM to NBD-TSO, while at of 0.5% (0.07 M), 1% (0.14 M), and 5% (0.7 M) concentration, DMSO diminished the NBD-TSO concentration about 5, 10 and 35%, respectively. In case of the other probes tested (CBA, FLBA, APC, and APF), the addition of even 0.05% v/v (7 mM) DMSO led to a strong inhibition of the fluorescence signal (Fig. 6). The presence of 0.5% v/v (0.07 M) or higher concentrations of DMSO was sufficient to completely scavenge HOCl and block the fluorescence increase.

## Discussion

In this study, several fluorogenic probes for HOCl were compared in terms of their applicability in screening and characterization of MPO inhibitors. Among the probes studied, two probes, i.e. CBA and FLBA contained boronate moieties as HOCl sensors, two probes possessed the *p*-aminophenyl moiety undergoing *O*-dearylation, i.e. APC and APF, and the NBD-TM probe based on the 7-nitrobenz-2-oxa-1,3-diazol fluorophore containing a HOCl-sensitive thiomorpholine substituent. Here, we report an optimized experimental setup for the detection of the HOCl-producing activity of MPO by those probes in the presence of H_2_O_2_ (Fig. 3).

The selectivity of the studied probes has been previously tested towards a broad range of various one- and two-electron oxidants, like ^•^OH, O_2_^•−^, H_2_O_2_, ONOO^−^, HOCl, etc.^36,38,39,44^ It has been shown that probes containing *p*-aminophenyl moiety as well as NBD-TM are not reactive toward H_2_O_2_ in the absence of a catalyst^39,44^. In turn, boronate probes react directly with H_2_O_2_, HOCl, and ONOO^−^, but the reaction rate constants vary over six orders of magnitude for those oxidants^29,36–38^. In fact, the rate constants of the reactions between boronates and H_2_O_2_ or boronates and HOCl are in the order of magnitude of 10^0^ and 10^4^ M^−1^s^−1^, respectively^29,36–38^. Consequently, this great difference in the reactivity of both species allows for the continuous monitoring of HOCl production in the MPO/H_2_O_2_/Cl^−^ system by boronate probes in the presence of low micromolar concentrations of the probes and H_2_O_2_ (Fig. 3).

APF is widely used in kits for determining the chlorination activity of myeloperoxidase and was used in this study as a reference probe. Both boronate probes reacted with HOCl at similar rates and performed in a similar manner to the APF probe (Fig. 3). The major difference was a slow oxidation of the boronate probes by H_2_O_2_ that was not observed in case of the APF probe. However, H_2_O_2_-dependent oxidation of boronates significantly contributed to the fluorescence signal at longer incubation time scales and was responsible for less than 10% of the signal at 20 min of incubation. This clearly indicates that boronate probes can be successfully used as alternative probes to APF for MPO-derived HOCl. In turn, APC detects lower amounts of HOCl in the MPO/H_2_O_2_/Cl^‒^ system in comparison to the CBA probe (Fig. 3a,c) suggesting the occurrence of side reactions in this system and/or probe interference with the enzymatic HOCl-production. Xiong et al. have shown that COH undergoes oxidative decomposition in the presence of HOCl^50^, but the results obtained for the CBA probe indicate that when the probe is present in excess of the oxidant, CBA is the major scavenger of HOCl (Fig. 3a). Thus, the oxidation of COH by HOCl in the presence of an excess of CBA or APC is unlikely because both probes are present in at least fivefold excess over the possible amount of COH formed and they react with HOCl with relatively high rate constants, expected to be orders of magnitude higher than for the reaction of COH with HOCl (Fig. 4). Interestingly, in the absence of sodium chloride, APC but not APF is also oxidized by MPO probably in the peroxidation cycle (Fig. 3c). This cannot, however, explain the lowered signal in comparison to the CBA probe (Fig. 3a,c) as at the high concentration of Cl^‒^, the MPO enzyme is completely switched to the chlorination cycle. Therefore, the potential reason of the lower signal observed for the APC probe is the influence of APC on the MPO activity. The results for NBD-TM are in agreement with the previous study^44^.

COH, fluorescein, and NBD-TSO were also studied in the MPO/H_2_O_2_/Cl^‒^ system to evaluate their stability under the established MPO assay conditions (Supplementary Figure S1). The most stable fluorescent signal among them was observed for fluorescein indicating that probes based on this fluorophore are superior to the COH or NBD-TM based probes (Supplementary Figure S1). Nonetheless, as long as an excess of probe is present in the reaction mixture, oxidation of the fluorophore will be inhibited. The optimized assay conditions included 20 µM concentration of the probes and the flux of HOCl was *ca.* 5 nM/s indicating that the steady-state concentration of MPO-derived HOCl was low and the level of the probe was in excess of both HOCl and probe-derived fluorescent products, over the entire time of incubation.

Thiols and thioethers are the biologically relevant scavengers of HOCl. Their reaction rate constants with HOCl are in the range of 10^7^–10^8^ M^−1^s^−1^^46,51,52^. The second biologically relevant scavengers of HOCl are amines with the second-order rate constants in a range between *ca.* 10^1^–10^6^ M^−1^s^−1^^46,53^. The rate constants found for HOCl and CBA, FLBA, APC, APF probes of 10^4^ M^−1^s^−1^ order of magnitude (Fig. 4) indicate that these probes may not effectively compete for HOCl intracellularly due to the more rapid reactions of HOCl with endogenous thiols and amines. However, in the absence of HOCl scavengers, their reactivity towards HOCl is sufficient for their use in the enzymatic assays for determining the chlorination activity of MPO and to study MPO inhibitors. NBD-TM that exhibits higher reactivity towards HOCl and reacts three orders of magnitude faster than the other probes tested, and at least one order of magnitude faster than amines and comparably fast to other biological thiols and thioethers, is expected to perform as an effective detector of HOCl also in cellular systems.

The rate constants determined for the boronate probes are consistent with those previously determined for this class of compounds^38,45,54,55^. The rate constants for APF and APC are also in agreement with the literature values for aromatic amines^46,53^. In turn, the rate constant for NBD-TM (^2^*k* = 1 × 10^7^ M^−1^s^−1^) is in reasonable agreement with the reaction rate constant of thioether L-methionine and HOCl (^2^*k* = 3.4 × 10^7^ M^−1^s^−1^)^41^.

The developed assays were used to determine the IC_50_ values for ABAH and the azide anion and the values obtained using different probes are convergent, but some deviations are observed (Table 1). These can be caused by specific interactions of the probes and/or products with MPO or other side reactions. Nonetheless, the FLBA, APF, and NBD-TM probes yielded the IC_50_ value of *ca.* 40 nM for ABAH and this value is close to the IC_50_ value determined by Amplex Red for the chloride free incubation (Table 1). For azide anion, the results were also convergent and close to the IC_50_ value obtained by the peroxidation activity assay (Table 1). Interestingly, IC_50_ values for the azide anion are more consistent between different probes, bringing up the possibility of specific interference of the coumarin-based probes or the COH product with the inhibition pathway of MPO by ABAH. ABAH is a suicide inhibitor that causes irreversible heme destruction of MPO and azide is a heme poison that exhibits a greater affinity for heme iron than oxygen causing permanent heme blockage of MPO^1,3^. Thus, both lead to the inhibition of the chlorination and peroxidase activity of MPO. Moreover, azide, which bonds to heme iron and inhibits formation of Compound I, should affect chlorination and peroxidase cycle in similar degree. In the case of ABAH, the IC50 values for both activities, excluding the results for the coumarin based probes, are in the reasonable agreement. The slight difference in the observed IC_50_ values for both cycles is probably due to the more complex inhibition mechanism^1,3,49,56^.

The Z’ parameter is a quantitative measure of the quality of an assay and indicates whether the developed assay is suitable for high-throughput screening applications^57^. The determined Z' values indicate that all studied assays are of good quality and can be used in both, end-point and kinetic mode.

We have also examined the effect of DMSO, often used to dissolve organic compounds intended for cell culture studies, on detected HOCl concentrations. As most chemical libraries involve the solutions of the compounds in DMSO, the resistance of the assay to small amounts (up to 0.1%, 14 mM) of DMSO is a condition to be met for its application in an HTS campaign. Among the studied probes, NBD-TM was the most resistant probe to the DMSO additive (Fig. 6). The data for other probes show that CBA, FLBA, APC, APF cannot be used in the presence of DMSO (Fig. 6). These results are in agreement with the determined rate constants (Fig. 4) and show that even a small addition of the DMSO solvent can have tremendous impact on the observed results and should be used with care when detecting reactive species such as HOCl.

It is also worth to discuss the use of absorption assays based on chlorination of taurine, like the TNB and TMB assays. The TNB assay that is based on the disappearance of a colored product can accurately measure concentrations of HOCl as low as 5 µM^22^. A more sensitive assay based on iodide catalyzed oxidation of TMB in the presence of taurine chloramine is five times more sensitive^23^. Substitution of TMB with dihydrorhodamine increases the sensitivity of this assay about 10 times shifting the HOCl detection limit to hundreds of nanomoles^23^. Similar limits of detection exhibit NBD-TM and other fluorescent probes (approx. 70–100 nM)^44^. Taking into account the rapid reaction between taurine and HOCl (^2^*k* = 4.8 × 10^5^ M^−1^s^−1^)^52^ the assays based on formation *N*-chlorotaurine may be sufficient for many purposes, where HOCl detection is needed. However, a requirement of the use of developing reagents and/or the need of a time-dependent probing of a reaction mixture to continuously monitor the MPO activity, in combination with their lower sensitivity in comparison to fluorescent assays (Supplementary Table S1) make these assays unsuitable for high-throughput screening experiments. In the case of TMB assay, the low stability of the signal should also be considered (Supplementary Figure S3). In turn, the assays based on the direct reaction of HOCl or OCl^‒^ with profluorescent compound are more sensitive (Supplementary Table S1) and enable a direct monitoring of the MPO chlorinating activity in time via a one-step mechanism, with high sensitivity avoiding the requirement of any developing reagent. Additionally, using the probe with strictly determined stoichiometry, e.g. boronate probes^38^, the absolute concentration of MPO-derived HOCl can be determined.

In the screening studies, where the large libraries of the potential inhibitors are investigated, usually more than one assay is used in order to exclude the false positive and false negative hits^28,31^. The use of two probes with different mechanisms of reaction with HOCl but with the same fluorescent product greatly simplifies the screening workflow. Due to the use of DMSO as a solvent in chemical libraries, we recommend the application of the NBD-TM probe in the primary screen. For confirmatory assays, where the compounds may be dissolved in solvents lacking HOCl-scavenging properties, we recommend the use of the APF and FLBA based assays, due to their high Z’ values, high sensitivity, and good stability of fluorescein in MPO/H_2_O_2_/Cl^‒^ system (Supplementary Figure S1).

## Conclusions

This study provides a comparison of three different classes of fluorescent probes for HOCl, with the goal of establishing a platform for high throughput screening of compounds in search of new inhibitors of MPO. We demonstrate that all three classes of the probes (boronate-, aminophenyl- and thiomorpholine-based fluorogenic probes) can be used for monitoring the chlorinating activity of MPO, but based on the IC_50_ values for ABAH we conclude that the probes containing coumarin moiety are not reliable indicators of inhibitors potency. The rate constants of the reaction between studied probes and HOCl were determined and the thiomorpholine-based NBD-TM probe proved to be the most rapid scavenger of HOCl. High reactivity of NBD-TM towards HOCl makes NBD-TM the most resistant probe to the addition of organic solvents, e.g. DMSO, a property that makes the probe applicable for HTS campaigns based on HOCl detection. We also propose to use fluorescein-based boronate (FLBA) and aminophenyl (APF) probes for the secondary/orthogonal assays in such HTS campaigns.

## Methods

### Used compounds and syntheses

MPO from human neutrophils was obtained from Athens Research and Technology (Athens, GA, According to the Certificate of Origin, the material used has been obtained from an FDA licensed collection center. Further the donors has given by signature full informed consent for the material to be used in commercial scientific research). Catalase form *Corynebacterium*, hydrogen peroxide, sodium hypochlorite, 4-aminobenzoic acid hydrazide (ABAH), NaN_3_, umbelliferone (COH), fluorescein, *N,N,N’,N’*-tetramethylbenzidine, NaCl, taurine, 5,5’-dithiobis(2-nitrobenzoic acid) (DTNB) were purchased from Sigma-Aldrich and were of the purest grade available. Fluorescent probes were synthetized according to the procedures described in the literature. The synthesis of fluorescein pinacol boron ester (Fl-B) was performed based on the procedures published by Dickinson et al. and Rios et al.^58,59^. The obtained boronate was then hydrolyzed to fluorescein boronic acid (FLBA). The synthesis of APF was performed based on the procedure published by Setsukinai and cooworkers^39^. The APC probe was synthesized according to the procedure published by Kavani and cooworkers^60^. The detailed description of synthetic procedures can be found in the Supplementary Information. The CBA synthesis is described elsewhere^37,61^.

### Plate reader measurements

The one millimolar stock solutions of CBA, and APC were prepared in acetonitrile. In the case of FLBA and APF, the two millimolar probe solutions were prepared in the water/acetonitrile mixture (1:1 v/v). Such prepared stock solutions were stable at least one-month and stored ready to use at 6 °C in the dark. The concentration of probes was checked during the day of plate reader measurements using an UV–Vis spectrophotometer (Varian Cary 300 Conc) and the determined molar absorption coefficients (Table 2).

**Table 2:** Absorption maxima (λ_max_), extinction coefficients (ε), fluorescence excitation (λ_exc_), and emission (λ_em_) maxima for the studied probes and their oxidation products.

Compound	UV–visible absorption		Fluorescence	
	λ_max_ [nm]	*ε* [M^−1^cm^−1^] (pH 7.4)	λ_exc_ [nm]	λ_em_ [nm]
NBD-TM	350^**a**^ 500^**a**^	7.8 × 10^3**a**^ 2.2 × 10^4**a**^		
NBD-TSO	485^**a**^	2.33 × 10^4**a**^	485	550
CBA	287^**b**^	1.2 × 10^4**b**^		
APC	324	1.6 × 10^4^		
COH	324^**b**^	1.3 × 10^4**b**^	332	450
FLBA	456	1.8 × 10^4^		
APF	455	2.35 × 10^4^		
FL	490	7.3 × 10^4^	490	515

^a^Reference^44^; ^b^Reference^35^

The lyophilized powder of MPO was dissolved in distilled water accordingly to the supplier recommendation and, if required, stored at 6 °C by the day of analysis. Such prepared solution was then diluted with phosphate buffer, pH 7.4, to the appropriate concentration and the volume of 0.5–2 µL/well depending on the activity was pipetted directly to 96-well plate (Costar 3840). Then, the enzyme was further diluted on a plate with volume of 100 µl of already prepared solutions containing phosphate buffer (100 mM) and other necessary components depending on the experiment or control, such as NaCl (0.2 M), the selected probe (40 µM) or taurine (20 mM), and catalase (200 U/ml). Reactions were stared adding 100 µl/well of 20 µM hydrogen peroxide solution. The concentration of hydrogen peroxide stock solution was determined spectrophotometrically at 240 nm using the molar absorption coefficient equal to 43.6 M^−1^cm^−1^. The changes of fluorescence intensity were measured by Varioscan LUX (Thermo Fisher Scientific) equipped with double monochromators based on diffraction gratings and controlled by SkanIt Software 6.0.2. The second plate reader used in the measurements was CLARIOstar (BMG Labtech) equipped with Linear Variable Filter (LVF) monochromators.

### Kinetic measurements

Kinetic measurements of the reaction of boronate probes, APC, and APF with HOCl were performed using a SX20 stopped-flow spectrometer (Applied Photophysics) equipped with 150 W Mercury-Xenon arc lamp. During the monitoring of COH formation the excitation monochromator was set at 330 nm and the emission monochromator was set at 450 nm. The formation of fluorescein was followed using the excitation wavelength of 492 nm and emission wavelength of 518 nm. During measurements, the slits width in the excitation and emission monochromators were set at 2.5 mm. The measurements were carried out under the pseudo-first-order conditions, where the concentration of a probe was at least five times higher than the concentration of HOCl. The observed pseudo-first-order rate constants were determined by the fitting of a curve described by the equation below (Eq. 1) to the kinetic traces measured at the chosen wavelength. Using the linear dependence of the pseudo-first-order rate constants on the initial concentration of a probe the appropriate second-order rate constants were determined.

1 $${\text{y }} = {\text{ y}}_{0} \left( {{1 }{-}{\text{ exp}}\left( {{\text{k}} \cdot {\text{t}}} \right)} \right)$$

In order to determine the second-order rate constant for the reaction of NBD-TM with HOCl the competition kinetic method was used and the CBA probe was exploited as a competitor. In this approach the two pseudo-first-order reactions are assumed. One reaction between NBD-TM and HOCl (Eq. 2) and the other between CBA and HOCl (Eq. 3). During the experiment the concentration of HOCl (0.5 µM) was three times lower than the concentration of NBD-TM (1.5 µM) and at least ten times lower in the case of the presence of CBA (50–900 µM). Due to the large difference in reactivity of CBA and NBD-TM toward HOCl and the limited solubility of both probes in the buffer solution, we were unable to use a higher concentration of NBD-TM to assure a higher excess of NBD-TM over HOCl. However, due to the excess of both NBD-TM and CBA over HOCl the changes in their concentration should have negligible effect on the determined rate constant. Under these conditions, at the constant concentration of NBD-TM, the yield of a specific product NBD-TSO was dependent on the concentration of CBA. Thus, using the determined rate constant (^2^*k*_CBA+HOCl_ = 1.8 × 10^4^ M^−1^s^−1^) and concentrations of the formed NBD-TSO, determined by LC/MS, the unknown second-order rate constant for the NBD-TM/HOCl reaction was calculated according to the equation below (Eq. 4)^32,34^.

2 $$CBA+HOCl\xrightarrow{{k_{{CBA+HOCl}}}}COH+B\left({OH}\right)_{3}+Cl^{-}$$

3 $$NBD-TM+HOCl\xrightarrow{{k_{{NBD-TM+HOCl}}}}+NBD-TSO+Cl^{-}$$

4 $$\frac{1}{{[NBD - TSO]}} = \frac{1}{{[NBD - TSO]_{0} }} + \frac{1}{{[NBD - TSO]_{0} }}\frac{{k_{{CBA + HOCl}} [CBA]}}{{k_{{NBD - TM + HOCl}} [NBD - TM]}}$$

LC/MS analyses were performed using an Acquity UPLC (Waters Ltd.) system combined with a LCT Premier XE (Waters Ltd.) mass spectrometer and a reversed-phase C_18_ UPLC column (Waters Acquity UPLC BEH C_18_ 1.7 mm, 50 × 2.1 mm) equilibrated with an acetonitrile/water mobile phase containing 0.1% trifluoroacetic acid (TFA). The samples containing NBD-TM and CBA, and their oxidation products after mixing with HOCl were separated using a flow rate of 0.3 ml/min and a gradient method with the initial concentration of the organic phase equal to 20%. This concentration was kept constant for 0.5 min after the sample injection. Then, the linear increase of acetonitrile from 20 to 100% over 1.5 min was applied.

### Z’ parameter

The Z’ factor is a dimensionless coefficient and was calculated on the basis of data presented in Fig. 3 according to the equation below (Eq. 5)^57^.

5 $$Z^{\prime} = 1 - \frac{{\left( {{\text{3S}}.{\text{D}}._{{{\text{control}} + }} - {\text{3S}}.{\text{D}}._{{{\text{control - }}}} } \right)}}{{\left( {{\text{mean}}_{{{\text{control + }}}} - {\text{mean}}_{{{\text{control - }}}} } \right)}},$$

where S.D._control+_ and S.D._control-_ are the standard deviation values, and mean_control+_ and mean_control-_ are the mean values for the positive and negative control, respectively. The value of Z ' above 0.5 means good quality of the assay. The higher value of Z ' parameter means the better quality of the assay. This parameter cannot reach value higher than 1.

## Supplementary Information

Supplementary Information.
