# Supplementary material for: Fluorescent probes for monitoring myeloperoxidase-derived hypochlorous acid: a comparative study
Source: Sci Rep. 2022 Jun 3;12:9314. doi: 10.1038/s41598-022-13317-8 (PMC9166712; doi:10.1038/s41598-022-13317-8)
Supplement: Supplementary file 1 — Supplementary Information. [file 41598_2022_13317_MOESM1_ESM.pdf]

Fluorescent probes for monitoring myeloperoxidase-derived hypochlorous acid – a comparative study

Karolina Pierzchała,[1] Marlena Pięta,[1] Monika Rola,[1] Małgorzata Świerczyńska,[2] Angelika Artelska,[1] Karolina Dębowska,[1] Radosław Podsiadły,[3] Jakub Pięta,[1] Jacek Zielonka,[3,*] Adam Sikora,[1] Andrzej Marcinek[1], and Radosław Michalski,[1,*]

[1]Institute of Applied Radiation Chemistry, Department of Chemistry, Lodz University of Technology, Zeromskiego 116, 90-924 Lodz, Poland

[2]Institute of Polymer and Dye Technology, Department of Chemistry, Lodz University of Technology, Stefanowskiego 12/16, 90-924 Lodz, Poland.

[3]Department of Biophysics and Free Radical Research Center, Medical College of Wisconsin, 8701 Watertown Plank Road, Milwaukee, WI 53226, United States

Corresponding author

*Radosław Michalski, PhD

Tel.: +48426313097

E-mail: radoslaw.michalski@p.lodz.pl

*Jacek Zielonka, PhD, DSc

Tel.: 001 414 955 4789

E-mail: jzielonk@mcw.edu

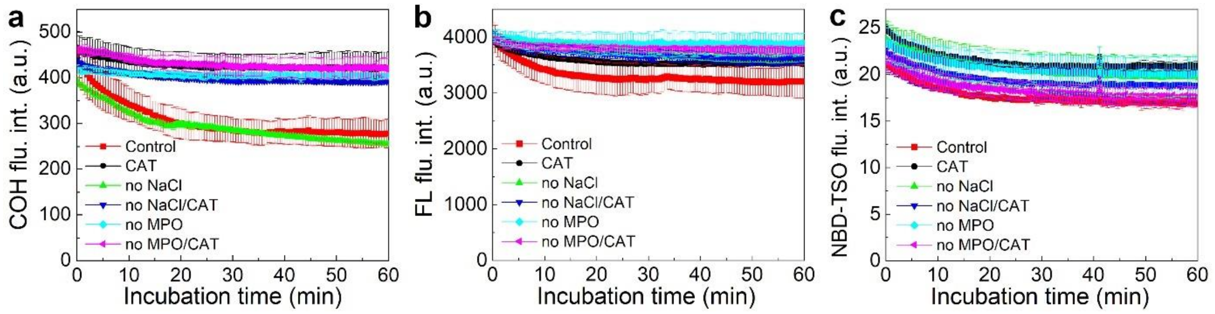

Supplementary Figure S1. (**a**) Decrease of COH fluorescence in the incubation mixtures containing COH (5 µM), MPO (1.2 nM), H_2O_2 (10 µM), NaCl (0.1 M), and phosphate buffer (50 mM, pH 7.4) (red); in the presence of catalase (100 U/ml, black); in the absence of NaCl (green); in the absence of NaCl but in the presence of catalase (dark blue); in the absence of MPO (light blue); in the absence of MPO but in the presence of catalase (pink). (**b**), (**c**) same as (**a**) but instead of COH, incubation mixtures contained fluorescein and NBD-TSO, respectively.

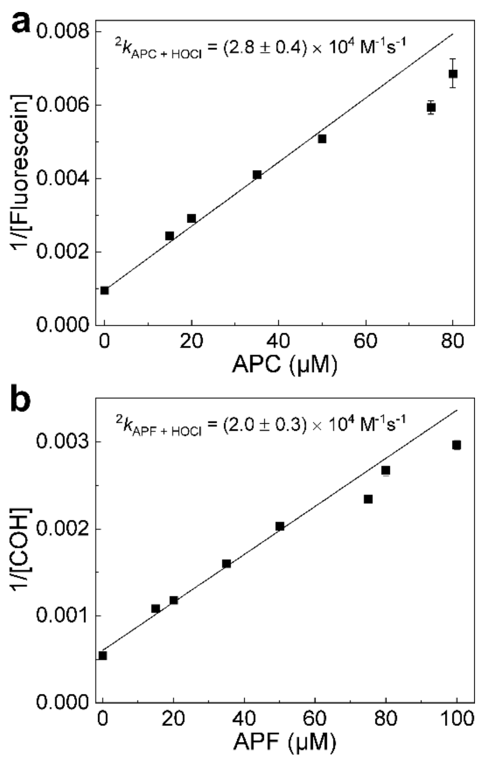

Supplementary Figure S2. The relationship used to determine the rate constant between APF and HOCl (**a**), or APF and HOCl (**b**), according to the competition kinetic approach. The solid lines in the panel (**a**) and (**b**) represent the linear fittings to the equation $\frac{1}{[fluorophore]} = \frac{1}{[fluorophore]_0} + \frac{1}{[fluorophore]_0} \frac{k_{APC/or\,APF}\,[APC/orAPF]}{k_{boronate}\,[boronate]}$. In the case of reaction between APC and HOCl (**a**), the set of incubation mixtures contained 50 mM phosphate buffer (pH 7.4), the APC probe (from 0 µM to 80 µM), HOCl (5 µM), and FLBA (25 µM) as a reference compound ($^2k_{FLBA+HOCl}$ = 1,11 × 10^4 M^{-1}s-^{1}). In the case of APF, the samples contained APF (0 – 100 µM), HOCl (5 µM), and CBA (25 µM) as a reference compound ($^2k_{CBA+HOCl}$ = 1,80 × 10^4 M^{-1}s-^{1}). The concentrations of COH and fluorescein were determined by LC/MS.

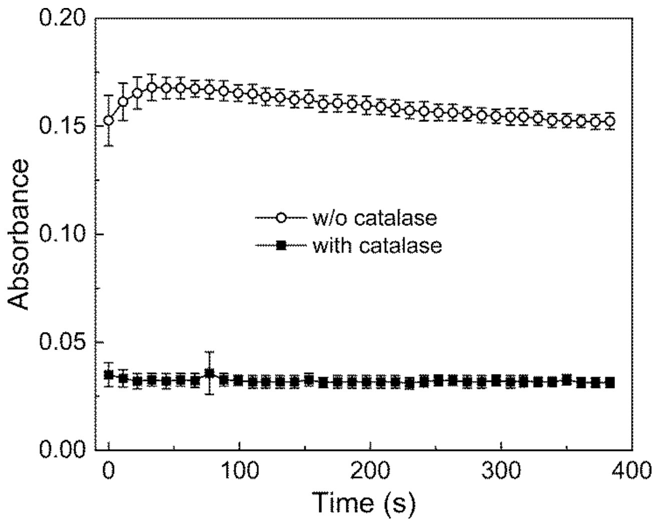

Supplementary Figure S3. Changes in the absorbance at 645 nm for the reaction mixture containing 0.4 mM 3,3',5,5'-tetramethylbenzidine, 0.08 mM acetate buffer, 20 µM potassium iodide and N-chlorotaurine. The solution of N-chlorotaurine was prepared in the incubation mixture containing 10 mM taurine, 2.75 mU/ml MPO, 10 µM H_2O_2, 0.1 M NaCl, and 20 mM phosphate buffer, in the presence (closed squares) or absence (open circles) 100 U/ml of catalase. The chlorination of taurine was stopped by addition of catalase (100 U/ml). Then, the solution of N-chlorotaurine was mixed with the solution containing 2 mM TMB, 100 µM potassium iodide, 0.4 M acetate buffer in 4:1 (v/v) ratio.

Determination of detection limits for CBA, APC, APF, FLBA, and NBD-TM

The detection limits were determined using the standard deviations of the blanks (σ) for each probe (CBA, APC, FLBA, APF, NBD-TM) and slopes of titration curves (s), according to the equation:

$$\text{Detection limit} = \frac{3.3\,\sigma}{s}$$

Titration curves were prepared measuring the fluorescence intensity for the incubations containing the chosen probe and various concentrations of HOCl. The standard deviation of the blank (σ) was calculated on the basis of the fluorescence intensity measured for the incubations containing of the selected probe but in the absence of HOCl.

Table S1. Detection limits determined for CBA, APC, APF, FLBA, and NBD-TM.

Probe	LOD
CBA	1.46 nM
APC	1.85 nM
APF	0.23 nM
FLBA	6.11 nM[a]
NBD-TM	99.65 nM

[a]The used batch of FLBA contained residual amounts of fluorescein, therefore the sensitivity may be improved by more extensive purification of the probe.

Syntheses

^{1}H NMR spectra were recorded on Bruker Avance II instrument at 700 MHz. Column chromatography was performed on Merck® silica gel 60 (70 – 230 mesh). Thin-layer chromatography was performed with precoated TLC sheets of silica gel 60 F 254 (Merck®). Preparative thin-layer chromatography was performed on precoated glass TLC plates of silica gel 60 with fluorescent indicator (Merck®). Reagents and starting materials were purchased from commercial vendors and used without further purification. Standard syringe techniques were used for transferring dry solvents.

Synthesis of 7-(4-aminophenoxy)-2H-chromen-2-one 4

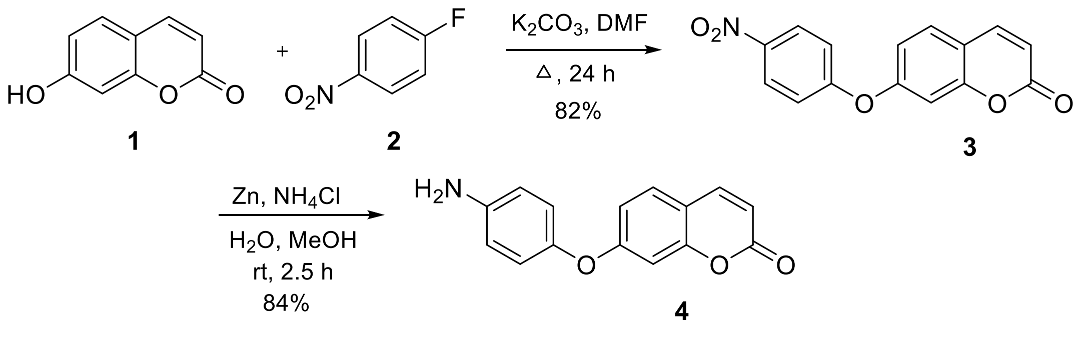

Synthesis was performed based on procedure published by Kavani and cooworkers.[1] A mixture of 7-hydroxy-2H -chromen-2-one **1** (1.23 mmol, 200 mg), 1-fluoro-4-nitrobenzene **2** (1.23 mmol, 174 mg), and K_2CO_3 (1.23 mmol, 170 mg) in anhydrous DMF (3 mL) was heated at 80°C for 24 h. The reaction mixture was cooled to room temperature and poured into crushed ice. The precipitated product was filtered and washed with cold water. Then it was dried and triturated with hexane to give 285 mg of 7-(4-nitrophenoxy)-2H-chromen-2-one **3** (82 % yield). ESI-MS m/z $[M+H]^+$ = 284.06. 7-(4-nitrophenoxy)-2H-chromen-2-one **3** (0.883 mmol, 250 mg) and NH_4Cl (5.30 mmol, 283 mg) were suspended in the mixture of H_2O (2 mL) and MeOH (12 mL) at room temperature. Then powder zinc (8.83 mmol, 577 mg) was added in small portions (within 15 min). It was stirred for 2.5 h and then filtered through celite cake. The solid was washed with MeOH (30 mL). The filtrate was concentrated, and the residue dissolved in AcOEt (20 mL) and H_2O (15 mL). Organic fraction was dried over Na_2SO_4 and concentrated to give 188 mg of pure 7-(4-aminophenoxy)-2H-chromen-2-one **4** as beige solid (84% yield). ^1H NMR, (700 MHz, DMSO-6d) δ 8.01 (d, *J* = 9.5 Hz, 1H), 7.66 (d, *J* = 8.6 Hz, 1H), 6.88 (dd, *J* = 8.6, 2.4 Hz, 1H), 6.87 – 6.84 (m, 2H), 6.75 (d, J = 2.4 Hz, 1H), 6.66 – 6.63 (m, 2H), 6.32 (d, J = 9.5 Hz, 1H), 5.20 (s, 2H). ESI-MS m/z $[M+H]^+$ = 254.08.

Synthesis of 2-[6-(4'-Amino)phenoxy-3H-xanten-3-on-9-yl]benzoic acid (APF)

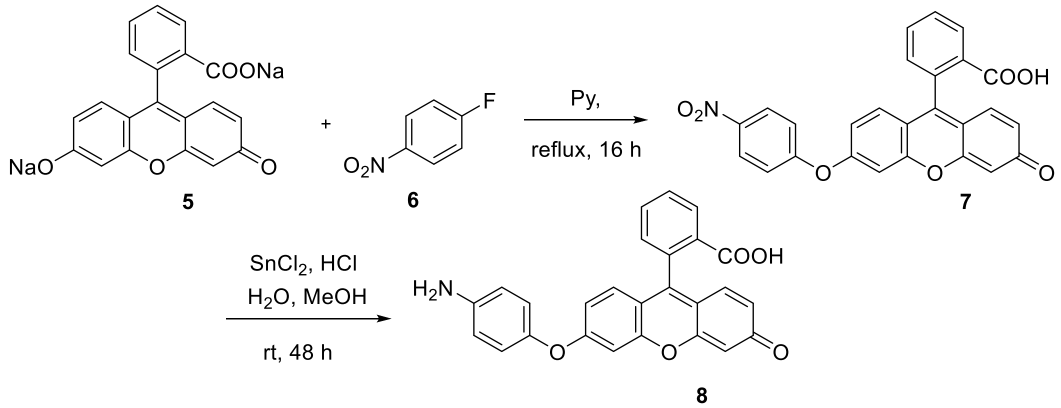

Synthesis was performed based on procedure published by Setsukinai and cooworkers.[2] Uranine **5** (400 mg, 1.06 mmol) and 4-fluoronitrobenzene **6** (600 mg, 4.26 mmol) were placed in the screw cap glass tube and dissolved in anhydrous pyridine (6 mL). The mixture was purged with argon and stirred in the oil bath set at 120 °C for 18 hours. Then it was cooled to rt and acidified with 4 M HCl to pH ca. 6. The mixture was diluted with H_2O and extracted with AcOEt. The organic layer was dried with Na_2SO_4, concentrated, and separated by column chromatography (hexane/AcOEt gradient from 95:5 to 50:50). 65 mg of desired compound **7** was obtained (13.5 % yield). ESI-MS m/z $[M+H]^+$ = 454.09. Then to 2-[6-(4'-nitro)phenoxy-3H-xanten-3-on-9-yl]benzoic acid **7** (65 mg, 0.143 mmol) suspended in conc. HCl*aq*/MeOH/H_2O (8 ml, 2:3:3) tin (II) chloride (72 mg, 0.301 mmol) was added. Additional $SnCl_2$ (144 mg, 0.602 mmol) was added portion wise over 22 hours. After additional 26 hours the mixture was basified with sat. $NaHCO_{3aq}$ to pH ca. 8-9. Then it was extracted with AcOEt, dried over Na_2SO_4 and condensed. The residue was purified by column chromatography ($CHCl_3$/AcOEt from 99:1 to 87:13). Obtained crude was then purified by preparative thin layer chromatography (CH_2Cl_2/MeOH, 97.5 : 2.5) to give 34 mg of 2-[6-(4'-amino)phenoxy-3H-xanten-3-on-9-yl]benzoic acid **8** as yellowish solid (56 % yield). ^1H NMR, (700 MHz, , MeOD) δ 8.04 – 8.01 (m, 1H), 7.76 – 7.79 (m, 1H), 7.73 – 7.69 (m, 1H), 7.21 – 7.23 (m, 1H), 6.89 – 6.86 (m, 2H), 6.81 – 6.78 (m, 2H), 6.74 – 6.70 (m, 2H), 6.65 -6.67 (m, 2H), 6.61 – 6.62 (m, 1H), 6.54 – 6.56 (m, 1H) ppm. ESI-MS m/z $[M+H]^+$ = 424.10.

Synthesis of Fluorescein boronic acid (FLBA)

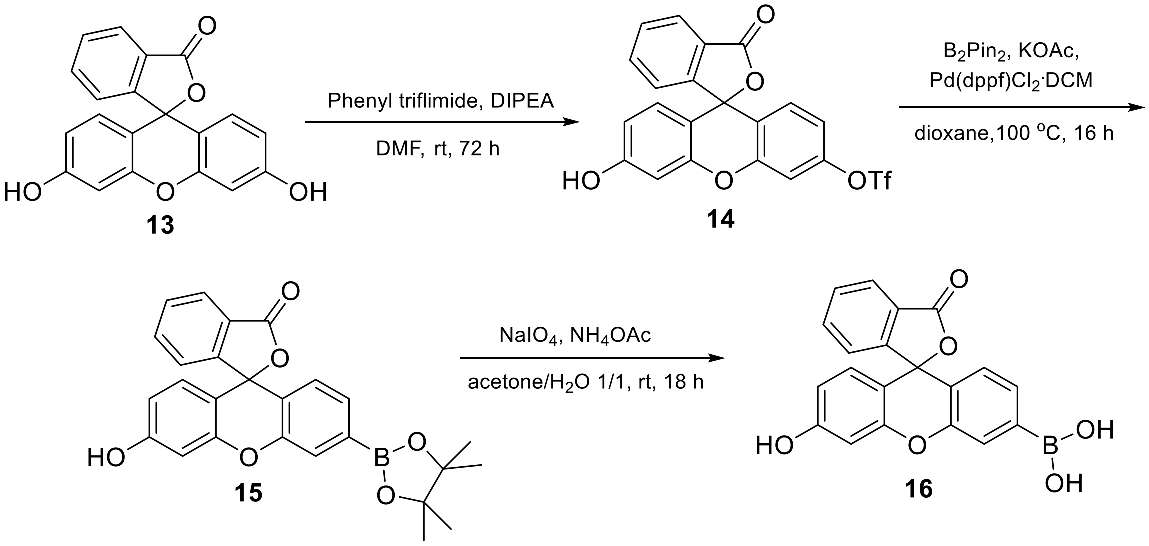

Two first steps of synthesis were performed based on procedure published in literature.[3] Fluorescein triflate **14** was obtained in reaction of fluorescein **13** (1.0 g, 3.01 mmol), phenyl triflimide (1.08 g, 3.01 mmol) and DIPEA (diisopropylethylamine) (2.0 mL, 14.3 mmol) in DMF (10 mL) under argon atmosphere with protection from light ensured. After acidification with 1 M HCl (10 mL) the mixture was extracted a few times with AcOEt, dried over $MgSO_4$ and condensed. Purification by column chromatography (hexane/AcOEt gradient from 100:0 to 91:9) gave 1.40 g of product **14** (65 % yield). ESI-MS m/z $[M+H]^+$ = 465.03. To obtain fluorescein pinacol boronester **15** suspension of triflate **14** (900 mg, 1.94 mmol), bis(pinacolato)diboron (700 mg, 2.76 mmol), Pd(dppf)Cl_2*CH_2Cl_2 (160 mg, 0.196 mmol) and KOAc (634 mg, 6.12 mmol) in dioxane (5 mL) was purged with argon for 20 min and stirred at 100°C. Cooled mixture was then diluted with AcOEt, washed with water and brine, dried over Na_2SO_4 and concentrated. Purification by column chromatography (hexane/AcOEt gradient from 95:5 to 70:30) gave 600 mg of pinacol boronate ester **15** (70 % yield). ESI-MS m/z $[M+H]^+$ = 443.16. Fluorescein boronic acid **16** was obtained in reaction of the ester **15** (300 mg, 0.678 mmol), $NaIO_4$ (1.16 g, 5.43 mmol) and NH_4OAc (418 mg, 5.43 mmol) in acetone and H_2O (25 mL, 1/1 vol/vol). Work-up consisted of evaporation of acetone and several extractions of the residue with AcOEt. Combined organic fractions were dried over $MgSO_4$ and concentrated. Purification by column chromatography ($CHCl_3$/MeOH gradient from 95:5 to 90:10) led to the desired product **16** in 86 % yield (210 mg, orange powder). Obtained product was then re-purified by preparative thin layer chromatography (PTLC) as follows. **16** was dissolved in a small volume of DCM and placed on the starting line (1.5 cm from bottom of the plate) of a PTLC glass plate (Merck, silica gel 60 Å matrix, 1000 µm, 20 x 20 cm with fluorescent indicator) with a Pasteur pipette as a narrow stripe. The PTLC was developed in DCM:MeOH (98:2 v/v) solvent system in a glass tank. The band containing FLBA was scraped from the plate with a metal spatula, transferred to the round bottom flask equipped with a magnetic stir bar. Around 50 cm3 of a DCM:MeOH (75:25 v/v) mixture was added and the suspension was mixed for 10 minutes after which it was filtered through a filter funnel. Solvents were evaporated under reduced pressure and the re-purified **16** was dried under high vacuum for 2 hours. ^1H NMR, (700 MHz, CD_3OD) δ 7.75 (t, *J* = 7.3 Hz, 1H), 7.71 (t, *J* = 7.3 Hz, 1H), 7.53 (s, 1H), 7.29 (dd, *J* = 6.8 Hz, 1H), 7.20 (d, *J* = 7.6 Hz, 1H), 6.76 (d, *J* = 7.4 Hz, 1H), 6.72 (d, *J* = 2.3 Hz, 1H), 6.61 (d, *J* = 8.7 Hz, 1H), 6.55 (dd, *J* = 8.7, 2.3 Hz, 1H). ESI-MS m/z $[M+H]^+$ = 361.09.

References

1. Kaviani, R.; Saeedi, M.; Mahdavi, M.; Nadri, H.; Moradi, A.; Shafiee, A.; Akbarzadeh, T. *Turk. J. Chem*. **2017**, *41*, 335-344.
2. Setsukinai, K.; Urano, Y.; Kakinuma, K.; Majima, H.J.; Nagano, T. *J. Biol Chem*. **2003**, *31*, 3170-3175.
3. Dickinson, B. C.; Huynh,C.; Chang, C. J. *J. Am. Chem. Soc*. **2010**, *132*, 5906–5915.
